# Crocodyliform Feeding Traces on Juvenile Ornithischian Dinosaurs from the Upper Cretaceous (Campanian) Kaiparowits Formation, Utah

**DOI:** 10.1371/journal.pone.0057605

**Published:** 2013-02-27

**Authors:** Clint A. Boyd, Stephanie K. Drumheller, Terry A. Gates

**Affiliations:** 1 Department of Geology and Geological Engineering, South Dakota School of Mines and Technology, Rapid City, South Dakota, United States of America; 2 Department of Geoscience, The University of Iowa, Iowa City, Iowa, United States of America; 3 Department of Earth and Planetary Sciences, The University of Tennessee, Knoxville, Tennessee, United States of America; 4 Department of Biology, North Carolina State University, Raleigh, North Carolina, United States of America; 5 Natural History Museum of Utah, Salt Lake City, Utah, United States of America; Raymond M. Alf Museum of Paleontology, United States Of America

## Abstract

Crocodyliforms serve as important taphonomic agents, accumulating and modifying vertebrate remains. Previous discussions of Mesozoic crocodyliform feeding in terrestrial and riverine ecosystems have often focused on larger taxa and their interactions with equally large dinosaurian prey. However, recent evidence suggests that the impact of smaller crocodyliforms on their environments should not be discounted. Here we present direct evidence of feeding by a small crocodyliform on juvenile specimens of a ‘hypsilophodontid’ dinosaur from the Upper Cretaceous (Campanian) Kaiparowits Formation of southern Utah. Diagnostic crocodyliform bite marks present on a left scapula and a right femur, as well as a partial probable crocodyliform tooth crown (ovoid in cross-section) preserved within a puncture on the right femur, comprise the bulk of the feeding evidence. Computed tomography scans of the femoral puncture reveal impact damage to the surrounding bone and that the distal tip of the embedded tooth was missing prior to the biting event. This is only the second reported incidence of a fossil crocodyliform tooth being found embedded directly into prey bone. These bite marks provide insight into the trophic interactions of the ecosystem preserved in the Kaiparowits Formation. The high diversity of crocodyliforms within this formation may have led to accentuated niche partitioning, which seems to have included juvenile dinosaurian prey.

## Introduction

Even though crocodyliforms long have been known to create feeding traces and bone accumulations (e.g., [1]–[2]), detailed, actualistic studies of modern crocodylian bite marks have only recently received attention [3]–[6]. Prior identifications and discussion of crocodylian and crocodyliform bite marks were often short and anecdotal, relying heavily on general comparisons of mark and tooth shape [7]–[9]. In the absence of positively-identified bite marks, discussion of crocodyliform trophic interactions have also been approached in terms of perceived morphological viability [10] or simple association of crocodyliform teeth with the remains of other vertebrate taxa [11]–[12].

More in-depth discussions of crocodyliform bite marks often took the form of paleontological differential diagnoses, in which the authors eliminated potential trace makers based on morphological, biomechanical, and ecological arguments. This technique often relied on isolated modern observations of feeding behavior or forensic case studies, and has been used to both exclude [13] and propose [14]–[16] specific crocodyliforms as the trace makers in paleontological and paleoanthropological contexts. However, in the absence of diagnostic traces to support these identifications, the argument could still be made that marks interpreted in this manner could have been created by taxa that remain unidentified or undiscovered.

The first large-scale, actualistic study of crocodylian bite marks centered on *Crocodylus niloticus* and identified a number of novel feeding traces and damage patterns [3]. Since this initial study, the unique feeding traces and bite mark patterns described by Njau and Blumenschine [3] have been found for other living and extinct crocodylian and non-crocodylian crocodyliform taxa [4], [5], [17]–[19], [20], suggesting that they may be diagnostic of the clade as a whole.

Studies addressing Mesozoic crocodyliform feeding have often centered on the largest members of the clade, and especially on their interactions with equally large dinosaurian prey [17], [21]. However, the effects of smaller crocodyliforms on their environments should not be discounted. Here we present direct evidence of feeding by a small crocodyliform on juvenile specimens of a basal ornithopod dinosaur from the Upper Cretaceous (Campanian) Kaiparowits Formation of southern Utah (hereafter referred to as the ‘Kaiparowits hypsilophodontid’).

### Geologic setting and locality information

The Kaiparowits Formation is exposed predominantly within the Grand Staircase-Escalante National Monument and seems to represent a wet, subhumid climate lowland fluvial depositional system [22] that was rich in both terrestrial and freshwater vertebrate taxa [23], [24]. The ∼860 m thick formation is divided into three informal units (lower, middle, and upper [22]), with the middle and lower units producing the highest abundance fossil material. The Kaiparowits Formation has been radiometrically age bracketed between 76.1 Ma and 74.0 Ma (^40^Ar/^39^Ar dating [25]), and the fossiliferous zones, including those of the middle unit, are roughly time correlative to other famous, vertebrate bearing formations (e.g., Dinosaur Park Formation of southern Alberta) that occurred along the Western Interior Basin [24], [25].

**Figure 1:**
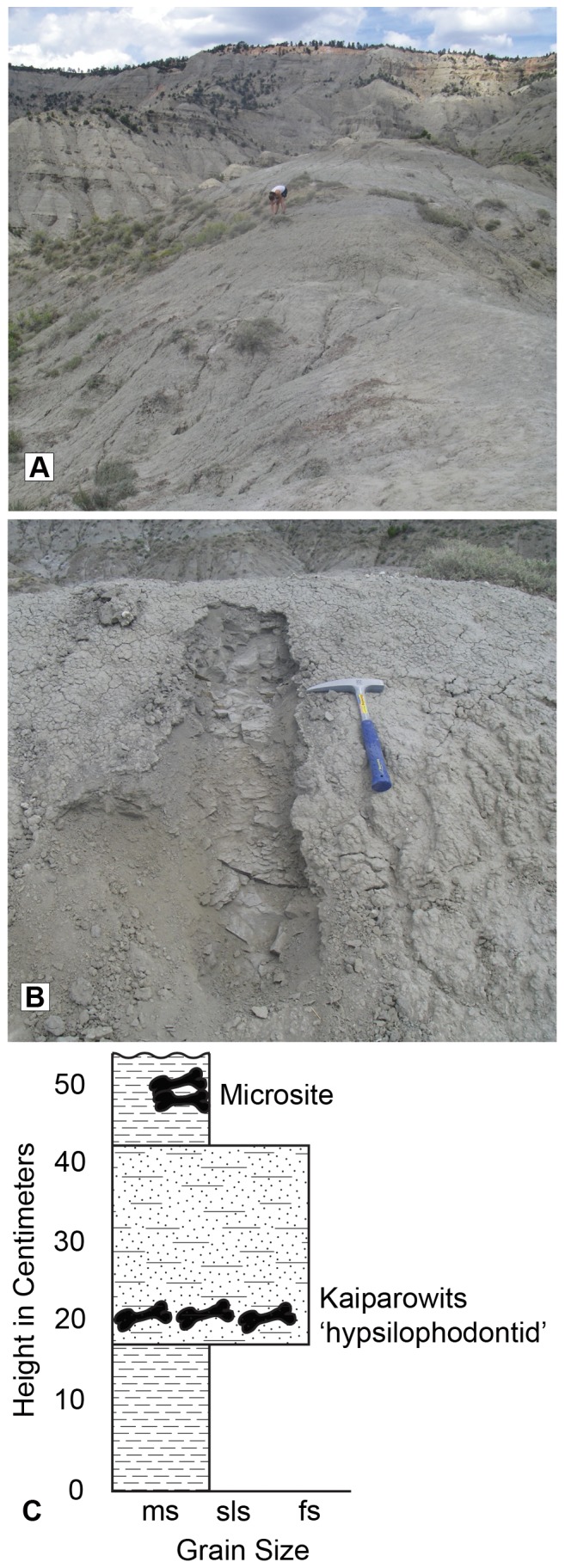
UMNH Locality 303 within the Kaiparowits Formation where UMNH VP 21104 and 21107 were collected. A. Overview photograph of the locality, which is located along the top of the ridge in the foreground. B. Photograph of microstratigraphic section through the locality. C. Grain size stratigraphic diagram through UMNH locality 303 showing the change to larger clasts within the bone layer as well as the presence of a microsite positioned above the bone layer. **Abbreviations: fs**, fine sandstone; **ms**, mudstone; **sls**, siltstone.

Discovered in 2002, UMNH (Natural History Museum of Utah) Locality 303 preserves the remains of at least three juvenile ‘hypsilophodontid’ ornithopods that were collected from around 400 m above the base of the formation, well within the middle unit, and approximately 20 m below a bentonite bed dated to 75.02±0.15 Ma (KBC 109: [24]). The site is located on the top of a large ridge within “The Blues” outcrop (contact UMNH for further information about this locality). Grain size increases in the bone bearing horizon from a predominantly clay-sized mudstone that forms the upper and lower bound of the bone-horizon to a layer rich in sand and silt sized clasts surrounding the fossils (Figure 1). This sandy-siltstone bed can be traced laterally across several other ridges in the area and is interpreted as a crevasse-splay deposit. The vast majority of the bones were found within the freeze-thaw zone of the sediment and had lost all association with each other. Screen washing of ∼10 kg of sediment from the site revealed no additional ornithopod elements or remains from other vertebrate taxa.

## Materials and Methods

Specimens UMNH VP (Natural History Museum of Utah Vertebrate Paleontology Collections) 21104 and 21107 were collected under BLM permit number UT-S-00-009. All material was examined with permission at the Natural History Museum of Utah and were also generously provided on loan by M. Getty for study and description. No additional permits were required for the described study, which complied with all relevant regulations.

### Examination and description of feeding traces

**Figure 2:**
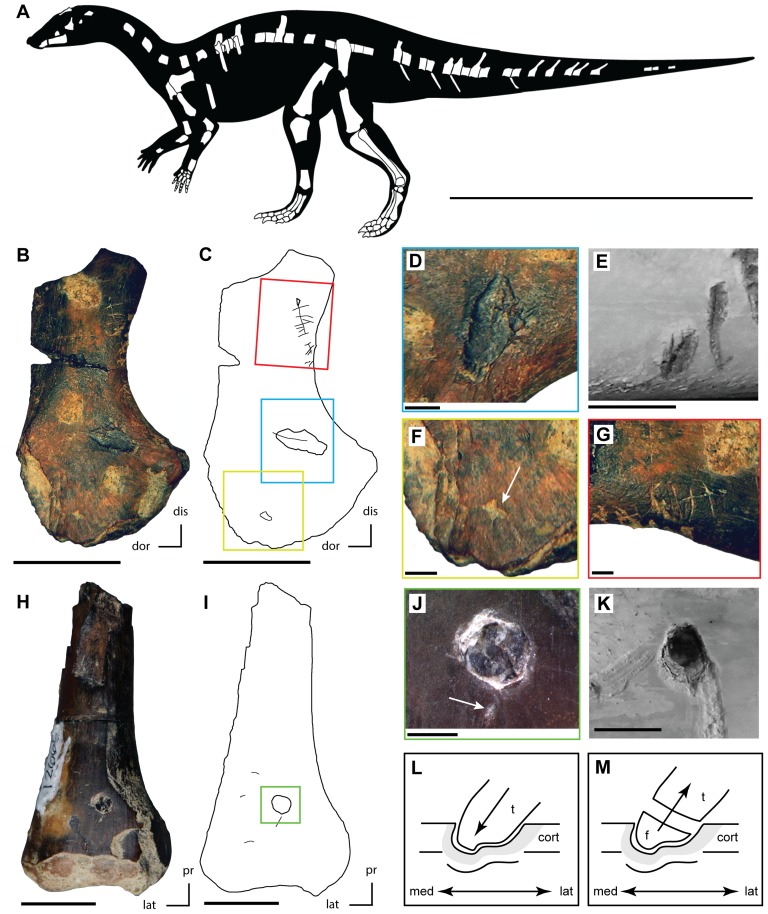
Feeding traces on juvenile ‘hypsilophodontid’ bones (Kaiparowits Formation) compared to those derived via actualistic experiments. A. Skeletal reconstruction of the undescribed ‘hypsilophodontid’ from the Kaiparowits Formation with known material shown in white (modified from [65]). B. Partial left scapula (UMNH VP 21104) with feeding traces collected from UMNH locality 303. C. Outline drawing of left scapula (UMNH VP 21104) with feeding traces highlighted and colored boxes showing the locations of figure parts D, F, and G (colors match the respective figure parts). D. Bisected pit on the left scapula (UMNH VP 21104). E. Bisected pit on a modern cow femur produced by *Alligator mississippiensis* during actualistic experiments [20]. F. Small pit (highlighted by white arrow) on the proximal portion of the left scapula (UMNH VP 21104). G. Series of small scores present along the ventral margin of the neck of the left scapula (UMNH VP 21104). H. Distal portion of a right femur (UMNH VP 21107) with feeding traces collected from UMNH locality 303. I. Outline of right femur (UMNH VP 21107) with feeding traces highlighted and colored box showing the location of figure part J. J. Puncture containing an embedded tooth present on the right femur (UMNH VP 21107) and a small pit (highlighted by white arrow) just ventral to the puncture. K. Puncture present on a modern cow femur produced by *A. mississippiensis* during actualistic experiments [20]. L. Reconstruction of the hypothesized impact of the crocodyliform tooth with the right femur, creating the puncture observed in UMNH VP 21107. M. Reconstruction of the hypothesized fracturing of the damaged crocodyliform tooth crown, resulting in the embedded tooth observed in UMNH VP 21107. Scale bar equals one meter in A, 10 mm in B, E, H, and K, 2 mm in D, F, G, and J. Abbreviations: cort, cortical bone; dis, distal; dor, dorsal; f, tooth fragment; lat, lateral; med, medial; pr, proximal; t, tooth crown.

Bite marks were identified using the method described by Blumenschine et al. [26] and categorized according to Binford’s [27] classification scheme (i.e. pits, punctures, scores, and furrows). Feeding traces were then compared to published descriptions of marks created by clades represented in the Kaiparowits Formation [3], [28]–[36], [37] and to a collection of modern bite mark specimens representing twenty-one crocodylian species [5], [20]. Potentially diagnostic patterns and structures (i.e. the bisected marks and hook scores described by Njau and Blumenschine [3]) were recorded and imaged (Figure 2).

### Computed tomography

One of the marks present on the ‘Kaiparowits hypsilophodontid’ was of particular interest because a tooth fragment was still embedded in the puncture. In order to further investigate this structure, the specimen was imaged in three dimensions using computed tomography (CT). The distal portion of a right femur referred to the ‘Kaiparowits hypsilophodontid’ (UMNH 21107) was scanned at the University of Texas High-Resolution X-ray CT Facility. The original CT data set consists of 475 consecutive slices having an interslice spacing and slice thickness of 0.02613 mm and an in-plane resolution of 0.02534 mm/pixel. These data are archived at the University of Texas High-Resolution X-ray CT Facility and available upon request.

## Results

### Description of prey species

The ‘Kaiparowits hypsilophodontid’ represents a previously undescribed taxon based on multiple autapomorphic traits that is closely related to the North American taxa *Oryctodromeus, Orodromeus,* and *Zephyrosaurus* based on the presence of direct pubosacral articulation (i.e., pubis rests against a facet on the sacral centra: [38]). Further, it shares with the latter two taxa the presence of a pronounced jugal boss [38]. Skeletal reconstructions indicate that the largest specimens present at UMNH locality 303 were roughly two meters in body length (Figure 2A), and estimates based on femoral circumference (largest specimens range from 63 to 75 mm) place this animal at 13 to 21 kg in mass [39]. The presence of largely unfused neurocentral sutures throughout the vertebral column and unfused sacral elements, partnered with preliminary histological investigations of associated material referred to this new taxon collected from UMNH locality 303, indicate that all specimens collected from this locality represent juvenile or subadult individuals [40].

### Description of bite marks – femur (UMNH VP 21107)

**Figure 3:**
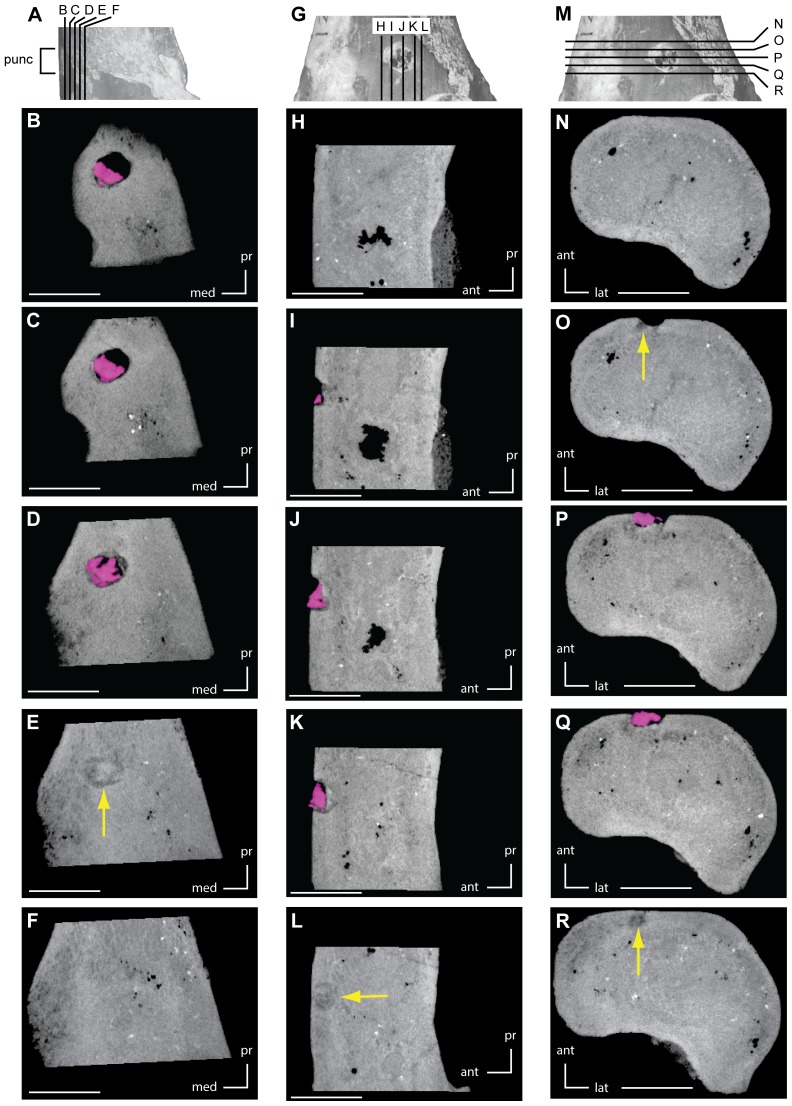
CT images of embedded tooth fragment and associated puncture on right femur (UMNH VP 21107). A. Right femur in medial view showing orientation of images B–F. B–F. CT images taken along the coronal plane, arranged in order from anterior to posterior. G. Right femur in anterior view showing orientation of images H–L. H–L. CT images taken along the sagittal plane, arranged in order from lateral to medial. M. Right femur in anterior view showing orientation of images N–R. N–R. CT images taken along the transverse plane, arranged in order from proximal to distal. Pink areas outline the embedded tooth fragment, while yellow areas indicate regions of compression damage to the surrounding bone. Scale bars equal 5 mm. **Abbreviations: ant**, anterior; **lat**, lateral; **med**, medial; **pr**, proximal; **punc**, puncture.

On the anterior surface of the right femur, just above the distal condyles, a partial conical tooth, ovoid in cross-section, is embedded in a puncture (Figure 2H, I, and J). The distal tip of the tooth is broken just below the outer surface of the bone, and it, as well as the associated puncture, is 2.5 mm in diameter. The distal end of the tooth truncates in a stepped fracture and the distal-most tip is completely missing, indicating that the tooth was broken prior to impacting this femur (Figure 3). These same scans reveal crushing of the bone internal to the puncture that indicates the already broken tooth contacted the anterior surface of the bone moving in a dorsolateral to ventromedial direction, at which point it fractured a second time, leaving the damaged crown embedded in the prey bone.

Two smaller pits occur between the puncture and the distal condyles (Figure 2I and J). One is just distal to the puncture and is 1.9 mm by 0.6 mm. The other is slightly more laterally positioned, occurring just proximal to the condyles, and is 1.7 mm by 0.6 mm. Two small scores are also present on the anterior surface of the femur, positioned laterally and proximally relative to the larger puncture (Figure 2I). Both are oriented roughly transversely to the long axis of the shaft and are 1.7 mm and 1.4 mm in length.

### Description of bite marks – scapula (UMNH VP 21104)

A large, elongate pit with a prominent bisection (*sensu* [3]) is present on the lateral surface, ventral to the scapular ridge and dorsal to the posterior edge of the glenoid fossa (Figure 2C and D). The bisection extends dorsally and curves slightly away from the main body of the pit. The entire mark is 2.0 mm in width and 5.5 mm in length. Anterior to this mark and also dorsal to the glenoid fossa is another small pit, 0.8 mm in diameter (Figure 2C and F).

A grouping of small scores, spanning an area of 12 mm, is located on the ventral edge of the base of the neck of the scapula (Figure 2G). Most are roughly 2 mm in length and are oriented transverse to the long axis of the scapular blade. Similar markings on the dorsal margin of this bone are largely obscured by sediment. A small pit is present just posterior to these scores and another distinct score extends from this pit anteriorly, crossing the other marks nearly perpendicularly (Figure 2C and G).

## Discussion

### Identification of trace maker

Taking the age and provenance of the fossils into account, there are three broad groupings of animals which could have created the larger of the two sets of bite marks seen on these bones: theropod dinosaurs, mammals or non-mammalian mammaliaforms, or crocodyliforms. There is an extensive literature documenting theropod feeding traces on a variety of dinosaur bones (e.g., [28]–[36]). The feeding traces on the ‘Kaiparowtis hypsilophodontid’ material lack striations, which are often caused by the serrations on ziphodont teeth, such as those possessed by theropods. This does not fully exclude theropods, because serrated teeth have been demonstrated to create un-striated bite marks [41]; however, the partial tooth crown embedded in the femur is ovoid in cross section. This tooth morphology is markedly different from the mediolaterally compressed teeth of most theropods [42]. Therefore, it is unlikely that theropod dinosaurs are the trace maker.

Some Cretaceous mammals were capable of preying on small dinosaurs (e.g., [43]). Again, the shape of the broken tooth embedded in UMNH VP 21107 does not resemble typical heterodont mammalian dentition, though it is possibly congruent with a broken tip of a caniniform tooth. However, the presence of the broken tooth itself would be highly unusual if the bite mark was made by a mammal. While crocodyliforms and theropods continually shed teeth throughout their lives, mammalian feeding behavior and tooth morphologies are at least partially driven by their inability to repeatedly replace old or damaged teeth. Even though it is possible that a mammalian caniniform tooth could be broken and embedded in prey bone owing to an injudiciously placed bite, this scenario does not reflect normal mammalian feeding behavior and tooth use [37]. The presence of a bisected pit goes further to rule out a mammalian trace-maker, as this type of bite mark is inconsistent with all published descriptions of mammalian bite marks [3].

Previous studies of crocodyliform bite marks on dinosaurian prey have often focused on large-bodied taxa, particularly *Deinosuchus* [17], [21], [44]–[45]. While bite marks from other crocodyliforms on dinosaurians are known [19], [46], the majority of examples come from other prey clades [8], [14]–[15], [47] such as turtles and mammals. The recent actualistic study involving *Crocodylus niloticus* [3] identified bite mark types and damage patterns created by modern crocodylians, including: 1) bisected pits and scores (diagnostic of crocodylians) and hook scores (diagnostic of taxa that utilize inertial feeding strategies [41]; 2) concentrations of feeding traces (> 10 marks) on major grasping areas (such as the neck of the scapula) resulting from attempts to disarticulate the skeleton into sections small enough to be swallowed; 3) a lower proportion of bones left by crocodyliforms bear feeding traces than those fed on by mammals (< 21% compared to > 50% [3]); 4) evidence of gnawing behavior is absent, which particularly differentiates crocodyliforms with their more restricted jaw mechanics from mammalians; and, 5) crocodyliforms typically leave whole bones or articulated skeletal units whereas mammalian carnivores tend to leave fragmented bones. These patterns have been found to be largely applicable to other extant and extinct crocodyliforms [3], [5], [17]–[19], [20].

The feeding traces on UMNH VP 21104 and 21107 and the condition of other associated material were evaluated using these six criteria to determine if they corresponded with either a mammalian or crocodyliform trace-maker. The presence of a bisected pit on the proximal scapula, the low frequency of bones recovered from UMNH locality 303 displaying feeding traces (< 10%), and the absence of gnawing on any broken margins or ends of long bones are consistent with a crocodyliform trace-maker. Evaluation of the fifth criterion is more difficult because this material was surface collected and not excavated in-situ. Most, if not all, observed bone fractures propagate perpendicularly to the long axis of long bones, which is characteristic of post-depositional, ‘dry’ fractures. This lack of green stick or spiral fracturing means that there is no evidence of breakage while the bone was still fresh, or ‘wet.’ Also, one set of metatarsals (II through IV) was articulated when collected and further articulation was possible at the time of initial deposition, as demonstrated by the roughly equivalent MNE (minimum number of elements) for long bones from the same skeletal regions. While tenuous, it is at least possible to state that the condition of the remains from UMNH locality 303 associated with specimens UMNH VP 21104 and 21107 is not inconsistent with criterion five.

The tooth fragment embedded in UMNH VP 21107 would have been roughly conical in shape prior to the loss of the tip and is ovoid in cross section, consistent with typical crocodyliform teeth. Despite the fact that modern crocodylians shed teeth throughout their lives, particularly during feeding [3], embedded teeth are quite rare. This specimen represents only the second report of a fossilized crocodyliform tooth found lodged directly in prey bone [48]. This tooth, partnered with the identification of a diagnostic crocodyliform feeding trace (the bisected pit) and other corroborating evidence (the placement and frequency of the bite marks) makes associating the larger bite marks (i.e., the puncture in the femur and the bisected pit on the scapula) with a crocodyliform trace maker possible.

As for the group of smaller bite marks on UMNH VP 21104, identification of the trace maker is more problematic. Rodents [49] and earlier mammalian taxa interpreted to have filled somewhat similar ecological niches [50] tend to create groups of subparallel or fan-shaped bite marks by gnawing on bone margins with their paired incisors. These marks are arranged together since they are created by repetitive, often overlapping bites from a small number of teeth. Such behavior is unlikely to have created the similar length, strongly parallel orientation, and nearly uniform spacing seen in all but two of the smaller bite marks present on UMNH VP 21104, implying instead that these were created by a series of teeth during a single biting event. If this is the case, then the similar cross-sectional profiles and spacing of these scores implies that the trace-maker had homodont rather than heterodont dentition. This excludes mammalians as the potential trace maker.

The smaller set of bite marks present on UMNH VP 21104 are not bisected, hooked (*sensu* [3]), or striated (*sensu* [41]). However, none of these types of bite marks were present in all, or even a majority, of sampled feeding traces created by crocodyliform teeth with prominent carinae or taxa with ziphodont dentition, such as theropods and some lizards. The concentration of at least fifteen scores on the base of the neck of the scapula (a major grasping point during disarticulation of the forelimb from the carcass) is consistent with criterion three from the Njau and Blumenschine study [3]. In the absence of other corroborating evidence, we cannot positively exclude crocodyliforms, theropods, or other small-bodied vertebrate groups with relatively homodont dentition as the trace maker for these small scores.

The Kaiparowits Formation preserves a particularly diverse crocodyliform faunal assemblage [51]–[53]. Two particularly large taxa have been identified, the gigantic alligatoroid *Deinosuchus riograndensis* ([52], [53]; *Deinosuchus hatcheri sensu* Irmis et al. [54]) and a possible goniopholidid or pholidosaurid [23], [54]. Adult individuals of these taxa can safely be excluded from consideration due to the size of the bite marks and embedded tooth fragment; yet, juvenile individuals cannot be summarily dismissed.

Other crocodyliforms that have been identified in the Kaiparowits Formation include *Brachychampsa* n. sp., either *Leidyosuchus* or *Borealosuchus* [53], [55], as many as four separate species of alligatoroid, including durophagous forms similar to *Allognathosuchus* or *Ceratosuchus* [51], [53], and a caimanine [51]. Many of these taxonomic assignments were based on highly fragmentary and/or undescribed material. A recent, apomorphy-based reanalysis of the Kaiparowits crocodyliforms limits this possible number of taxa considerably [54]. Many specimens previously identified as belonging to a particular genus or species were found to lack diagnostic features identifying them beyond broad taxonomic classifications (e.g., Crocodyliformes, Mesueucrocodylia, Neosuchia). The presence of a possible new species of *Brachychampsa* was verified, with a formal description of the material forthcoming [54]. At least one additional alligatoroid, distinct from *Brachychampsa*, was also identified, but multiple taxa may also be present. Irmis et al. [54] identified no conclusively durophagus forms. Even though the recent revision greatly clarifies Kaiparowits crocodyliform diversity at around four to five taxa, without the discovery of more complete specimens and further descriptions of existing specimens, eliminating any of these taxa as the potential trace maker remains difficult.

When attempting to further characterize the crocodyliform trace maker from a gross morphological instead of a systematic standpoint, the isolated, fragmented nature of the embedded tooth makes estimating vital statistics such as exact size of the entire animal difficult. However, the small minimum size of the tooth (2.5 mm in diameter) in rough comparison to modern crocodylian dentition (*Alligator mississippiensis*; CAB, pers. obs.) suggests a small individual, perhaps one meter in length. The lack of extensive secondary alterations, in the form of widespread crushing and fracturing related to the biting event [56], also points towards a smaller individual, since crocodyliform bite force has been shown to scale with body size [57]–[58]. Extant crocodylians between 1 and 1.8 m in length are known to prey on animals between five and twenty-five kg in mass [3], consistent with the estimated size of the ‘Kaiparowits hypsilophodontid’ individuals preserved at UMNH locality 303 (13–21 kg based on femur circumference [39]).

## Conclusions

Extant crocodylians are often apex predators in their respective environments, able to prey upon all but the largest terrestrial vertebrates. Given that some extinct Cretaceous crocodyliforms, such as *Deinosuchus* and *Sarcosuchus* [59], dwarf even the largest living crocodylians, it is reasonable to assume that these taxa were capable of killing and eating a variety of dinosaurian prey. A growing body of literature cites bite marks as direct evidence of trophic interactions between crocodyliforms and dinosaurians, utilizing evidence beyond body size and cranial morphology correspondence to prey items. Schwimmer [21] presented direct evidence in the form of possible feeding traces on dinosaurian bones that he attributed to *Deinosuchus*. However, in the absence of comparative data derived from actualistic studies, some questioned his association of the traces with feeding behavior at all, much less with *Deinosuchus* specifically [60]. With the publication of Njau and Blumenschine’s [3] diagnosis of modern crocodylian bite marks, subsequent studies of large crocodyliform bite marks on dinosaurian remains have had an actualistic foundation for comparison to support their interpretations (e.g., [17], [19]).

However, the possibility that smaller crocodyliform species, or even immature individuals of larger species, may have fed on dinosaurian prey has garnered significantly less attention, despite modern ecological studies demonstrating niche partitioning along prey size parameters related to size even within single species of modern crocodylians (e.g., [61]–[63]). The evidence of feeding by small crocodyliforms on juvenile dinosaurians presented here provides an opportunity to discuss crocodyliform feeding dynamics and paleobiology beyond the largest members of the group.

The Kaiparowits Formation has yielded an unusually high diversity of crocodyliforms, even though detailed descriptions have not yet been performed for all recovered taxa [51]–[54]. Roughly five taxa have been identified, including two particularly large-bodied species: *Deinosuchus* [52]–[53] and a possible goniopholidid or pholidosaurid [23], [54]. These juvenile and adult crocodyliforms would have inhabited the river systems of the region, preying on other aquatic and terrestrial taxa that also used the water as a resource. Modern crocodylian species whose geographical ranges overlap have been observed to segregate themselves with regards to their dietary preferences and spatial distributions within single ecosystems [64]. The Kaiparowits crocodyliforms would be expected to have reacted to similar pressures in their environment in a broadly comparable manner.

## Acknowledgments

We thank M. Getty for access to specimens at the Natural History Museum of Utah, the staff of the Saint Augustine Alligator Farm, especially D. Kledzik, for access to their animals, Southeastern Provisional and Swaggerty’s Farms for donating the modern samples for bite mark collection, the Jackson School of Geosciences at The University of Texas at Austin for allowing CAB access to facilities, C. Brochu, M. Householder, M. Stocker, and J. Horton the UI Paleontological Discussion Group for helpful support and comments, and the Utah BLM office (especially A. Titus) for assistance with collecting permits, access to BLM specimens, and general discussion on this project.
